# Flexible Host Choice and Common Host Switches in the Evolution of Generalist and Specialist Cuckoo Bees (Anthophila: *Sphecodes*)

**DOI:** 10.1371/journal.pone.0064537

**Published:** 2013-05-17

**Authors:** Jana Habermannová, Petr Bogusch, Jakub Straka

**Affiliations:** 1 Department of Zoology, Faculty of Science, Charles University in Prague, Praha, Czech Republic; 2 Department of Biology, University of Hradec Králové, Hradec Králové, Czech Republic; Australian Museum, Australia

## Abstract

Specialization makes resource use more efficient and should therefore be a common process in animal evolution. However, this process is not as universal in nature as one might expect. Our study shows that *Sphecodes* (Halictidae) cuckoo bees frequently change their host over the course of their evolution. To test the evolutionary scenario of host specialization in cuckoo bees, we constructed well-supported phylogenetic trees based on partial sequences of five genes for subtribe Sphecodina (Halictini). We detected up to 17 host switches during *Sphecodes* evolution based on 37 ingroup species subject to mapping analysis of the hosts associated with the cuckoo bee species. We also examine the direction of evolution of host specialization in *Sphecodes* using the likelihood ratio test and obtain results to support the bidirectional evolutionary scenario in which specialists can arise from generalists, and vice versa. We explain the existence of generalist species in *Sphecodes* based on their specialization at the individual level, which is recently known in two species. Our findings suggest flexible host choice and frequent host switches in the evolution of *Sphecodes* cuckoo bees. This scenario leads us to propose an individual choice constancy hypothesis based on the individual specialization strategy in cuckoo bees. Choice constancy has a close relationship to flower constancy in bees and might be an extension of the latter. Our analysis also shows relationships among the genera *Microsphecodes*, *Eupetersia*, *Sphecodes* and *Austrosphecodes*, a formerly proposed *Sphecodes* subgenus. *Austrosphecodes* species form a basal lineage of the subtribe, and *Microsphecodes* makes it paraphyletic.

## Introduction

If there were a species that could utilize all available resources equally well, it would certainly be the most successful species on Earth. In fact, there is no such species because adaptation to an extremely wide spectrum of different resources is impossible. According to Van Valen's Red Queen hypothesis [1], resource specialization seems to be a necessary condition to keep pace in an evolutionary race between interacting species (an “arms race” [2]). Apparently, there are two opposing pressures: it would be beneficial to utilize a broad resource spectrum, but specialization is necessary for effective resource utilization. Therefore, we can find resource specialists as well as resource generalists, and each strategy has some advantages and disadvantages.

Specialists could be favored over generalists because they do not face the problems of antagonistic adaptations to different resources; thus, specialists can respond to counter-adaptations more effectively [3]. They can also process information more easily during the search for suitable resources [4] and occupy a less competitive environment [5]. Finally, a new advantageous allele that allows more efficient resource utilization can spread faster in specialized species [6], [7].

Generalists, by contrast, could benefit from greater resource availability and therefore require less investment in resource acquisition [5], [8]. It is unclear whether the generalist strategy is simply a temporary condition in evolutionary terms that will inevitably lead to specialization as the possible stable strategy.

Alternatively, perhaps specialists are prone to extinction because their narrow adaptation inhibits the utilization of other resources [3]. According to this hypothesis, specialization is an evolutionary dead end [9]. Cospeciation, or “missing the boat” (i.e., species utilize only one of two new resources established through speciation) [10] are the routes through which specialists can switch to new resources. Switching to a new unrelated resource is impossible under these assumptions.

However, the dead-end hypothesis is not consistent with the results of many empirical studies, as summarized in Hoberg's and Brooks' review [11] of parasites. Host switches with no or weak cospeciation with hosts are also commonly documented in herbivorous insects [12], [13], [14], [15], [16], [17], [18]; thus, generalists can evolve from specialist species [14], [15]. A decreasing trend of host specificity has also been documented in parasites, such as fleas [19] or Monogenea [20].

The ecological fitting hypothesis explains the results of most empirical studies. According to this hypothesis, a common evolutionary history is not necessary for the mutual adaptation of two species. The reason why two species are adapted to each other results from the coincidence of their compatibility regarding important characters [21]. The switch to a new resource is conditioned by potential fitness, which the species would have in a new situation [22]. Another explanation reflects the fact that some resources do not require special adaptations for their utilization. Such conditions allow easy switches to new resources [23], especially switches between ecologically similar resources [11].

Resource specialization appears to be a very complex process, which is potentially shaped by many factors. An example is provided by pollen specialization, until now, the only well studied case of resource specialization in bees. There are both pollen generalists and pollen specialists [24]. Theoretically, the generalist strategy would be the more advantageous because it would allow all types of pollen resources to be utilized. Nevertheless, this strategy is not possible because the choice of host flower is physiologically or neurologically constrained. The pollen of certain plants may be toxic to some bee species, resulting in a physiological constraint [25], [26]. Even the larvae of generalist species are unable to develop on the pollen collected by another generalist species [27]. A neurological constraint arises because bees have limited memory and learning capacities with respect to shape and color [28], [4]. Therefore, the host flowers within bee species are usually similar [29]. A pollen specialist would, in theory, be better adapted to these conditions; however, pollen generalist species of bees exist, and the generalist strategy seems to be the derived condition [30], [31]. Generalist species may be successful because their pollen-collecting behavior resembles that of the specialist. Generalist species focus on a single host plant species before switching to new host flowers after a couple of foraging visits [32]. This phenomenon, called flower constancy, may allow bees to overcome neurological constraints [32]. Similarly, the specialized strategy could be more flexible than it may first appear: in several pollen specialized bee species, host switching has been observed in the absence of their usual flower host [33], [34]. Such bee species may have one essential host but be able to utilize other hosts.

Here, we address the cuckoo bees, a parasitic group of bees that do not collect pollen. Cuckoo bees neither build their own nest nor provision brood cells. Instead, these bees search for nests of other bee species and lay eggs in their cells. The host provisions of pollen are used as nutrition by the offspring of the cuckoo bee. Cuckoo behavior is relatively common in bees. Obligatory cuckoo species are found in four of the seven bee families [24], and 27 independent origins of this behavior are now identified in bees [24], [35], [36]. Most cuckoo bee species are specialists, having one or a few closely related hosts. Nevertheless, generalist species with many hosts of different genera and families are also known [37], [38]. We investigated the evolution of host specificity in the genus *Sphecodes* Latreille in this study because the host species are relatively well studied and both specialist and generalist species occur within this genus [39]. Moreover, two generalist *Sphecodes* species (*S. monilicornis* and *S. ephippius*) are specialized at the individual level: individual females repeatedly visit the nests of the same host species, but different females visit different hosts [38]. *Sphecodes* belongs to the tribe Halictini, subtribe Sphecodina, a group consisting of 249 species worldwide. These species live in all ecosystems and are most likely all parasitic [24]. *Microsphecodes* Eickwort and Stage, *Eupetersia* Blüthgen and *Ptilocleptis* Michener are the other genera of this subtribe [24]. Michener [40], [24] suspects that *Eupetersia* and *Ptilocleptis* are basal taxa of Sphecodina and recognizes two subgenera in *Sphecodes*: *Sphecodes* sensu stricto and *Austrosphecodes* Michener. Nevertheless, no phylogenetic study has been conducted to date, making the evolutionary relationships within this subtribe unclear.

Using the cuckoo bees as a model, this paper seeks to enhance our understanding of the general principles of resource specialization. Therefore, our goals were (1) to develop a robust phylogeny of the subtribe Sphecodina and discuss the result, (2) to test whether cuckoo bees of the genus *Sphecodes* have evolved in concert with their host species and whether host switches are rare or common, (3) to test whether generalism is an ancestral strategy in cuckoo bees and whether reversals in host specialization have occurred, (4) to determine whether specialization at the individual level is just a unique and relatively unstable strategy in cuckoo bee evolutionary history and whether this generalist strategy can lead to species diversification through specialization on new hosts and (5) to develop a hypothesis of resource specialization based on our results.

## Methods

### Materials and data sets

The complete data set comprises 107 specimens of 48 species from the subtribe Sphecodina and 20 species from the subfamilies Rophitinae, Nomiinae, Nomioidinae and Halictinae (tribes Augochlorini, Caenohalictini and Halictini) used as outgroups. Most of the sequences are newly developed for this study. The sequences of the outgroup taxa and some *Sphecodes* were acquired from the NCBI database (Table 1). A reduced data set including only one specimen per species and all outgroup species was used for our primary analysis and for the reconstruction of ancestral character states. The complete list of specimens, locality data and GenBank accession numbers are listed in Table 1. Voucher specimens of taxa used for the first time were deposited in the J. Straka collection (Charles University in Prague). Most bee specimens were preserved in 96% EtOH; several pinned specimens were also used. The nomenclature follows Michener [24] and Bogusch and Straka [39].

**Table 1:** Complete list of specimens, locality data and Genbank accession numbers of sequences.

			GenBank Accession numbers				
Taxon, No.	Collection country	Align1	28S	COI	EF1	LWR	WG
*Agapostemon tyleri*	–	x	AY654506.1^a^	AF102835.1^b^	AF140320.1^c^	AY227940.1^d^	AY222577.1^e^
*Augochloropsis metallica*	–	x	GU320093.1^f^	–	AF140315.1^c^	AY227934.1^d^	AY222571.1^e^
*Austrosphecodes* sp. 1	Peru	x	JX256745	JX256648	JX256448	JX256782	JX256546
*Austrosphecodes* sp. 12	Argentina	x	JX256746	JX256649	JX256449	JX256783	JX256547
*Austrosphecodes* sp. 13	Argentina	x	JX256747	JX256650	JX256450	JX256784	JX256548
*Austrosphecodes* sp. 14	Argentina	x	JX256748	JX256651	JX256451	JX256785	JX256549
*Conanthalictus wilmattae*	–	x	AY654508.1^a^	–	AF435378.1^g^	AY227916.1^d^	AY222553.1^e^
*Dieunomia nevadensis*	–	x	DQ060852.1^h^	–	AF435396.1^g^	AY227931.1^d^	AY222568.1^e^
*Dufourea novaeangliae*	–	x	–	FJ582211.1^i^	AF435384.1^g^	AY227919.1^a^	AY222556.1^e^
*Eupetersia seyrigi*	–	x	–	–	EU203259.1^e^	EU203287.1^e^	EU203228.1^e^
*Eupetersia* sp. 1	Angola	x	JX256749	JX256652	JX256452	JX256786	JX256550
*Halictus ligatus*	–	x	–	AF102840.1^b^	AF140299^c^	AY455895.1^j^	AY455899.1^j^
*Halictus quadricinctus*	–	x	–	AF438422.1^k^	AF140334.1^c^	AY227956.1^d^	AY222592.1^e^
*Lasioglossum calceatum*	–	x	–	AF103980.1^b^	AF435385.1^g^	AF448877.1^l^	AY222608.1^e^
*Lasioglossum florale*	–	x	–	AF103955.1^b^	AF264792.1^m^	AY227966.1^d^	AY222602.1^d^
*Lasioglossum hybodinum*	–	x	GU320096.1^f^	AF104660.1^b^	AF264857.1^m^	AY227963.1^d^	AY222599.1^d^
*Lasioglossum lustrans*	–	x	–	AF104643.1^b^	AF435388.1^g^	AF448904.1^l^	AY222609.1^e^
*Lasioglossum zephyrum*	–	x	–	AF103974.1^b^	AF435379.1^g^	AF448918.1^l^	AY222607.1^e^
*Lasioglossum zonulum*	–	x	–	AF104658.1^b^	AF264855.1^m^	AY227969.1^d^	AY222606.1^d^
*Microsphecodes* sp. 10	Venezuela	x	JX256750	JX256653	JX256453	JX256787	JX256551
*Microsphecodes* sp. 15	Bolivia	x	JX256751	JX256654	JX256454	JX256788	JX256552
*Nomioides facilis*	–	x	AY654511.1^a^	–	AF435394.1^g^	AY227929.1^d^	AY222566.1^d^
*Patellapis abessinica*	–	x	GU320097.1^f^	–	EU203267.1^e^	EU203295.1^e^	EU203236.1^e^
*Penapis penai*	–	x	AY654513.1^a^	–	AF435401.1^g^	AY227921.1^d^	AY222558.1^e^
*Ruizantheda mutabilis*	–	x	GU320094.1^f^	–	AF435406.1^g^	AY227949.1^d^	AY222586.1^e^
*Sphecodes albilabris* 1	Czech Republic	x	JX256752	JX256655	JX256455	JX256789	JX256553
*Sphecodes albilabris* 2	Czech Republic	–	–	JX256656	JX256456	–	JX256554
*Sphecodes albilabris* ssp. 3	Spain	–	–	JX256657	JX256457	–	JX256555
*Sphecodes alternatus* 1	Hungary	x	JX256753	JX256658	JX256458	JX256790	JX256556
*Sphecodes alternatus* 2	Italy	–	–	JX256659	JX256459	–	JX256557
*Sphecodes autumnalis*	–	x	–	FJ582459.1^i^	EU203256.1^e^	–	EU203225.1^e^
*Sphecodes* cf. *dusmeti*	Iran	x	JX256758	–	JX256468	JX256793	JX256566
*Sphecodes clematidis*	–	x	–	FJ582469.1^i^	EU203257.1^e^	–	EU203226.1^e^
*Sphecodes confertus*	–	x	–	FJ582472.1^i^	EU203258.1^e^	–	EU203227.1^e^
*Sphecodes crassus* 1	Czech Republic	x	JX256756	JX256663	JX256463	–	JX256561
*Sphecodes crassus* 2	Slovakia	–	–	JX256664	JX256464	–	JX256562
*Sphecodes crassus* 4	Czech Republic	–	–	JX256665	JX256465	–	JX256563
*Sphecodes crassus* 6	Switzerland	–	–	JX256666	JX256466	–	JX256564
*Sphecodes cristatus*	Slovakia	x	JX256757	JX256667	JX256467	JX256792	JX256565
*Sphecodes croaticus*	Czech Republic	x	JX256755	JX256662	JX256462	–	JX256560
*Sphecodes ephippius 1*	Hungary	x	JX256759	JX256668	JX256469	JX256794	JX256567
*Sphecodes ephippius* 2	Czech Republic	–	–	JX256673	JX256474	–	JX256572
*Sphecodes ephippius* 3	Czech Republic	–	–	JX256674	JX256475	–	JX256573
*Sphecodes ephippius* 4	Czech Republic	–	–	JX256675	JX256476	–	JX256574
*Sphecodes ephippius* 8	Czech Republic	–	–	JX256676	JX256477	–	JX256575
*Sphecodes ephippius* 9	Hungary	–	–	JX256677	JX256478	–	JX256576
*Sphecodes ephippius* 14	Italy	–	–	JX256669	JX256470	–	JX256568
*Sphecodes ephippius* 15	Iran	–	–	JX256670	JX256471	–	JX256569
*Sphecodes ephippius* 16	Bulgaria	–	–	JX256671	JX256472	–	JX256570
*Sphecodes ephippius* 17	Czech Republic	–	–	JX256672	JX256473	–	JX256571
*Sphecodes ferruginatus* 1	Czech Republic	x	JX256760	JX256678	JX256479	JX256795	JX256577
*Sphecodes ferruginatus* 2	Czech Republic	–	–	JX256679	JX256480	–	JX256578
*Sphecodes geoffrellus* 1	Czech Republic	x	JX256761	JX256680	JX256481	JX256796	JX256579
*Sphecodes geoffrellus* 2	Czech Republic	–	–	JX256681	JX256482	–	JX256580
*Sphecodes geoffrellus* 7	Czech Republic	–	–	JX256682	JX256483	–	JX256581
*Sphecodes gibbus* 1	Czech Republic	x	JX256762	JX256683	JX256484	JX256797	JX256582
*Sphecodes gibbus* 2	Czech Republic	–	–	JX256685	JX256486	–	JX256584
*Sphecodes gibbus* 3	Czech Republic	–	–	JX256686	JX256487	–	JX256585
*Sphecodes gibbus* 4	Slovakia	–	–	JX256687	JX256488	–	JX256586
*Sphecodes gibbus* 5	Czech Republic	–	–	JX256688	JX256489	–	JX256587
*Sphecodes gibbus* 6	Czech Republic	–	–	JX256689	JX256490	–	JX256588
*Sphecodes gibbus* 7	Czech Republic	–	–	JX256690	JX256491	–	JX256589
*Sphecodes gibbus* 10	Iran	–	–	JX256684	JX256485	–	JX256583
*Sphecodes hyalinatus* 3	Czech Republic	–	–	JX256691	JX256492	–	JX256590
*Sphecodes hyalinatus* 4	Bulgaria	x	JX256763	JX256692	JX256493	JX256798	JX256591
*Sphecodes longuloides*	Spain	x	JX256764	JX256693	JX256494	JX256799	JX256592
*Sphecodes longulus* 1	Slovakia	x	JX256765	JX256694	JX256495	JX256800	JX256593
*Sphecodes longulus* 2	Czech Republic	–	–	JX256695	JX256496	–	JX256594
*Sphecodes longulus* 3	Italy	–	–	JX256696	JX256497	–	JX256595
*Sphecodes majalis*	Czech Republic	x	JX256767	JX256703	JX256505	JX256803	JX256603
*Sphecodes marginatus* 6	Tunisia	–	–	JX256698	JX256499	–	JX256597
*Sphecodes marginatus* 7	Tunisia	–	–	JX256699	JX256500	–	JX256598
*Sphecodes marginatus* 10	Tunisia	x	JX256766	JX256697	JX256498	JX256801	JX256596
*Sphecodes miniatus* 2	Slovakia	–	–	JX256700	JX256501	–	JX256599
*Sphecodes miniatus* 4	Czech Republic	–	–	JX256701	JX256502	–	JX256600
*Sphecodes miniatus* 7	Switzerland	–	–	JX256702	JX256503	–	JX256601
*Sphecodes miniatus* 9	Slovakia	x	–	–	JX256504	JX256802	JX256602
*Sphecodes monilicornis* 1	Czech Republic	x	JX256768	JX256704	JX256506	JX256804	JX256604
*Sphecodes monilicornis* 2	Czech Republic	–	–	JX256706	JX256508	–	JX256606
*Sphecodes monilicornis* 4	Czech Republic	–	–	JX256707	JX256509	–	JX256607
*Sphecodes monilicornis* 7	Czech Republic	–	–	JX256708	JX256510	–	JX256608
*Sphecodes monilicornis* 8	Italy	–	–	JX256709	JX256511	–	JX256609
*Sphecodes monilicornis* 9 ssp. *cephalotes*	Turkey	–	–	JX256710	JX256512	–	JX256610
*Sphecodes monilicornis* 10	Bulgaria	–	–	JX256705	JX256507	–	JX256605
*Sphecodes niger*	Czech Republic	x	JX256769	JX256711	JX256513	JX256805	JX256611
*Sphecodes nomioidis* 1	Czech Republic	x	JX256770	JX256712	JX256514	JX256806	JX256612
*Sphecodes nomioidis* 3	Czech Republic	–	–	–	JX256515	–	JX256613
*Sphecodes olivieri*	Tunisia	x	–	JX256713	JX256516	–	JX256614
*Sphecodes pellucidus* 1	Slovakia	x	JX256771	JX256714	JX256517	–	JX256615
*Sphecodes pellucidus* 11	Czech Republic	–	–	JX256715	JX256518	–	JX256616
*Sphecodes pellucidus* 3	Czech Republic	–	–	JX256716	JX256519	–	JX256617
*Sphecodes pellucidus* 4	Switzerland	–	–	JX256717	JX256520	–	JX256618
*Sphecodes pellucidus* 5	Hungary	–	–	JX256718	JX256521	–	JX256619
*Sphecodes pinguiculus*	Cape Verde	x	JX256773	JX256720	JX256523	JX256807	JX256621
*Sphecodes pseudofasciatus*	Czech Republic	x	JX256772	JX256719	JX256522	–	JX256620
*Sphecodes puncticeps* 1	Hungary	x	JX256775	JX256723	JX256526	JX256809	JX256624
*Sphecodes puncticeps* 11	Morocco	–	–	JX256724	JX256527	–	JX256625
*Sphecodes ranunculi*	–	x	–	FJ582493.1^i^	AF140325.1^c^	AY227961.1^d^	AY222597.1^e^
*Sphecodes reticulatus* 2	Czech Republic	x	–	JX256726	JX256529	JX256811	JX256627
*Sphecodes reticulatus* 3	Italy	–	–	JX256727	JX256530	–	JX256628
*Sphecodes rubicundus*	Czech Republic	x	JX256776	JX256725	JX256528	JX256810	JX256626
*Sphecodes ruficrus* 1	Tunisia	x	JX256778	JX256728	JX256531	JX256812	JX256629
*Sphecodes ruficrus* 2	Spain	–	–	JX256729	JX256532	–	JX256630
*Sphecodes rufiventris* 1	Czech Republic	x	JX256779	JX256730	JX256533	JX256813	JX256631
*Sphecodes rufiventris* 2	Czech Republic	–	–	JX256731	JX256534	–	JX256632
*Sphecodes rufiventris* 3	Italy	–	–	JX256732	JX256535	–	JX256633
*Sphecodes rufiventris* 4	Czech Republic	–	–	JX256733	JX256536	–	JX256634
*Sphecodes scabricollis* 3	Germany	x	–	–	JX256544	–	JX256644
*Sphecodes scabricollis* 4	Germany	–	JX256781	JX256742	JX256545	JX256815	JX256645
*Sphecodes schenckii* 1	Turkey	x	JX256754	JX256660	JX256460	JX256791	JX256558
*Sphecodes schenckii* 2	Iran	–	–	JX256661	JX256461	–	JX256559
*Sphecodes* sp. 17	Canada	x	JX256780	JX256734	JX256473	JX256814	JX256635
*Sphecodes* sp. 18	Canada	–	–	JX256735	–	–	JX256636
*Sphecodes* sp. 2	Canada	–	–	JX256736	JX256538	–	JX256637
*Sphecodes* sp. 21	Japan	–	–	JX256737	JX256539	–	JX256638
*Sphecodes* sp. 26	–	–	–	AF102844.1^b^	AF140324.1^c^	AY227960.1^d^	AY222596.1^e^
*Sphecodes* sp. 3	Canada	–	–	JX256738	JX256540	–	JX256640
*Sphecodes* sp. 7	USA	–	–	JX256739	JX256541	–	JX256641
*Sphecodes* sp. 8	USA	–	–	JX256740	JX256542	–	JX256642
*Sphecodes* sp. 9	USA	–	–	JX256741	JX256543	–	JX256643
*Sphecodes spinulosus* 1	Czech Republic	x	JX256774	JX256721	JX256524	JX256808	JX256622
*Sphecodes spinulosus* 2	Hungary	–	–	JX256722	JX256525	–	JX256623
*Sphecodes zangherii* 2	Switzerland	x	–	JX256743	–	–	JX256646
*Sphecodes zangherii* 3	Switzerland	–	–	JX256744	–	–	JX256647
*Systropha curvicornis*	–	x	AY654516.1^a^	–	AF435411.1^g^	AY227925.1^d^	AY222562.1^e^
*Thrinchostoma lemuriae*	–	x	–	–	EU203254.1^e^	EU203285.1^e^	EU203223.1^e^
*Xeralictus bicuspidariae*	–	x	AY654517.1^a^	–	AF435413.1^g^	AY227927.1^d^	AY222564.1^e^

At sequences, which are obtained from NCBI database, links to the references are quoted: ^a^ Danforth et al. [78], ^b^ Danforth [79], ^c^ Danforth et al. [80], ^d^ Danforth et al. [81], ^e^ Danforth et al. [82], ^f^ Cardinal and Danforth (unpublished), ^g^ Danforth [83], ^h^ Danforth et al. [84], ^i^ Sheffield et al. [85], ^j^ Danforth and Brady (unpublished), ^k^ Soucy and Danforth [86], ^l^ Danforth et al. [87], ^m^ Danforth and Ji [88].

### Preparation of DNA sequences

DNA was extracted from part of the abdomen or from the entire individual and then homogenized. DNA was isolated using the Dneasy Blood & Tissue Kit (Qiagen) according to the manufacturer's protocol. Partial sequences of the following five genes were amplified by PCR: cytochrome oxidase subunit I (COI), elongation factor-1 alpha F2 copy (EF1), wingless (WG), long-wavelength rhodopsin (LWR) and 28S ribosomal RNA (28S). A list of the primers and PCR conditions is given in Table 2. The sequences were edited using the program 4Peaks 1.7.2 [41], then aligned in Clustal W and realigned manually using BioEdit 7.0.9 [42]. All of the alignments are available from www.aculeataresearch.com and www.treebase.org.

**Table 2:** List of primers including PCR conditions.

Locus	Primer	Orientation	Sequence 5′→3′	Reference
**COI** a				
	AP-J-1991	Forward	TAT AGT TAT ACC ATT TTA ATT G	modified from Pedersen [89]
	AP-J-2013	Forward	GGA GGA TTT GGA AAT TGG CTT ATT CC	modified from Simon et al. [90]
	AP-J-2511	Forward	GAA GTT TAT ATT TTA ATT TTA CCT GG	modified from Simon et al. [90]
	AP-N-2536	Reverse	CCA GGT AAA ATT AAA ATA TAA ACT TC	modified from Simon et al. [90]
	AP-N-2950	Reverse	GCA AAT ACA GCA CTT ATT GA	modified from Pedersen [89]
	AP-N-2980	Reverse	GGA WAT CCA TGA ATA AAT CTT G	unpublished
	Ron	Forward	GGA TCA CCT GAT ATA GCA TTC CC	Simon et al. [90]
	Pat	Reverse	TCC AAT GCA CTA ATC TGC CAT ATT A	Simon et al. [90]
**EF1** b				
	HaF2For1	Forward	GGG YAA AGG WTC CTT CAA RTA TGC	Danforth et al. [80]
	F2-Rev1	Reverse	A ATC AGC AGC ACC TTT AGG TGG	Danforth et al. [80]
**WG** c				
	beewgFor	Forward	TGC ACN GTS AAG ACC TGY TGG ATG AG	Danforth et al. [81]
	Lep wg2a	Reverse	ACT ICG CAR CAC CAR TGG AAT GTR CA	Danforth et al. [81]
**LWR** d				
	LWRhFor	Forward	AAT TGC TAT TAY GAR ACN TGG GT	Danforth et al. [81]
	LWRhRev	Reverse	ATA TGG AGT CCA NGC CAT RAA CCA	Danforth et al. [81]
**28S** e				
	28SD3for	Forward	GAC CCG TCT TGA AAC ACG GA	Schulmeister [91]
	28SD3rev2	Reverse	CCC ACA GCG CCA GTT CTG CTT ACC	Schulmeister [91]

a PCR conditions: 94°C 5 min, 40×(94°C 1 min, 37–50°C 1 min, 72°C 1 min 50 s), 72°C 5 min;

b PCR conditions: 94°C 5 min, 35×(94°C 1 min, 54–60°C 1 min, 72°C 1 min 50 s), 72°C 5 min;

c PCR conditions: 94°C 5 min, 35×(94°C 45 s, 58°C 45 s, 72°C 45 s), 72°C 5 min;

d PCR conditions: 94°C 5 min, 35×(94°C 1 min, 52–60°C 1 min, 72°C 1 min), 72°C 5 min;

e PCR conditions: 94°C 5 min, 35×(94°C 45 s, 52°C 45 s, 72°C 45 s), 72°C 5 min.

### Phylogenetic analysis

Two concatenated alignments were created using the web application FaBox 1.35 [43]. The main alignment (Align1) contains data from only the reduced dataset (one specimen per species) and sequences of all five genes for a total of 3,679 nucleotide sites (873 base pairs [bp] for COI, 1,152 bp for EF1 including a 394 bp long intron, 403 bp of WG, 632 bp of LWR including a 174 bp long intron and 619 bp of 28S). Align1 was used for the phylogenetic analysis, molecular dating and the reconstruction of ancestral character states. A supplementary alignment (Align2) contains data from the complete dataset but for only three genes (COI, EF1, WG), for a total of 2,428 nucleotide sites. Align2 was used for the supplementary phylogenetic analyses.

Bayesian and maximum likelihood (ML) analyses were performed for each concatenated alignment. Initially, several alignment subsets were created for each gene according to codon position, introns, exons or stem and loops. The best DNA substitution model was chosen for each subset and for the concatenated alignments based on the AICc value (the AIC value [44] corrected for sample size) [45], [46] using the program jModelTest 2 [47]. If the best model was not implemented in the MrBayes program [48], the best implemented model was chosen for Bayesian analysis. Subsequently, several Bayesian and ML analyses were performed with different partitioning schemata for each gene (no partitioning, intron/exon, intron/exon 1st +2nd/3rd exon, intron/exon 1st/2nd exon/3rd exon and stem/loop).

The results of the Bayesian analyses of all partitioning strategies for each gene were compared using Bayes factors [49]. The marginal likelihoods were estimated as the harmonic mean of the likelihood scores using the sump command in MrBayes. The statistic 2 ln(BF) was then calculated as 2[ln(HM1) – ln(HM2)], where HM1 is the harmonic mean of the posterior sample of likelihoods from the one partitioning strategy and HM2 is the harmonic mean of the posterior sample of likelihoods from the other partitioning strategy. According to Brandley et al. [50], values of 2 ln(BF) less than 2 indicate that the first partitioning strategy (HM1) is not significantly better than the second (HM2). Subsequently, the best partitioning strategy for each gene was determined. The results of the maximum likelihood analyses of all the partitioning strategies for each gene were compared using the AIC values [44]. The partitioning strategy with the lowest AIC was considered the best strategy.

Subsequently, three analyses of the concatenated alignments of different partitioning schemata were performed: (1) no partitioning, (2) the most partitioned schema (by the codon positions of each gene and stem and loop structures) and (3) the best partitioning schemata for each gene. The three partitioning strategies of the concatenated alignments were compared using the Bayes factor and the AIC value in the same way as the partitioning schemata of particular genes were compared. Finally, the best partitioning strategies for each method (Bayesian and ML) were determined.

All Bayesian analyses were conducted using MrBayes [48] implemented in BioPortal [51]. Each analysis consisted of running four simultaneous chains for 10 million generations and saving every thousandth tree. Two independent analyses starting from different random trees were performed for each partitioning strategy. The convergence of chains was inspected by checking the posterior distributions of log likelihoods using the program Tracer [52]. The first 25% of trees of each independent analysis were discarded as burn-in. A consensus tree was created from the best partitioned analyses according to the Bayes factor.

All of the ML analyses were calculated using the program Garli [53] implemented in BioPortal [51]. Four independent search replicates were performed for each analysis. One thousand bootstrap replicates were performed for calculating the branch support values of the best partitioned strategy (according to the AIC value) for Align1 and Align2. A bootstrap consensus tree was constructed with the program PAUP 4.0b10 [54].

### Definition of character states for character mapping and test of irreversible evolution

Two types of characters were mapped onto the phylogenetic tree of the genus *Sphecodes*: the ancestral host group and the ancestral host specificity (specialist or generalist). The list of 37 *Sphecodes* species with mapped characters is given in Table 3.

**Table 3:** List of mapped characters.

Taxon	Specialization	Hosts							
		LS	LO	H	S	A	C	P	M
*S. albilabris* (Fabricius)	(S) **G** (G)			x			x		x
*S. alternatus* Smith	**S**			x					
*S. autumnalis* Mitchella, b	**S**							x	
*S. clematidis* Robertson	?								
*S. confertus* Say*^c^*	**S**					x			
*S. crassus* Thomson	(S) **G** (G)		x						
*S. cristatus* Hagens	(S) **S** (G)		x		x				
*S. croaticus* Meyer	**S**		x						
*S. dusmeti* Blüthgen	?								
*S. ephippius* (Linnaeus)	**G**	x	x	x	x	x			
*S. ferruginatus* Hagens	**S**		x						
*S. geoffrellus* (Kirby)	(S) **G** (G)		x						
*S. gibbus* (Linnaeus)	(S) **G** (G)		x	x					
*S. hyalinatus* Hagens	**S**		x						
*S. longuloides* Blüthgen*^d^*	**S**		x						
*S. longulus* Hagens	**S**		x						
*S. majalis* Pérez	**S**	x							
*S. marginatus* Hagens	**S**		x						
*S. miniatus* Hagens	**S**		x						
*S. monilicornis* (Kirby)	**G**	x	x	x	x	x			
*S. niger* Hagens	**S**		x						
*S. nomioidis* Pesenko	?								
*S. olivieri* Lepeletier	**S**	x	x						
*S. pellucidus* Smith	(S) **G** (G)	x				x			
*S. pinguiculus* Pérez	**S**				x				
*S. pseudofasciatus* Blüthgen	?								
*S. puncticeps* Thomson	**S**		x						
*S. ranunculi* Robertson	?								
*S. reticulatus* Thomson	(S) **G** (G)	x				x			
*S. rubicundus* Hagens	**S**					x			
*S. ruficrus* (Erichson)	**S**					x			
*S. rufiventris* (Panzer)	**S**			x					
*S. scabricollis* Wesmael	**S**	x							
*S. schenckii* Hagens	**S**	x							
*S.* sp. 17	?								
*S. spinulosus* Hagens	**S**	x							
*S. zangheri* Noskiewicz	?								

The specializations are S, specialist and G, generalist. The state used in the main analysis (Distribution II) is shown in bold, that used in Distribution I is shown on the left in parentheses, and that used in Distribution III is shown on the right. The hosts are LS, *Lasioglossum* sensu stricto; LO, *Lasioglossum* other; H, *Halictus* subgenus *Halictus*; S, *Halictus* subgenus *Seladonia*; A, *Andrena*; C, *Colletes*; P, *Perdita*; and M, *Melitturga*. If unspecified, information on specialization and hosts is adopted from Bogusch and Straka [39].

a Eickwort [57], ^b^ Danforth [56], ^c^ Mike Arduser personal communication, ^d^ Blüthgen [55].

#### Ancestral host group

The list of known host species of *Sphecodes* used in our analyses was adopted from data in Bogusch and Straka [39], Michener [40], Blüthgen [55], Danforth [56] and Eickwort [57]. The hosts of *Sphecodes* species were divided into several groups according to their genera (*Andrena*, *Colletes*, *Perdita*, and *Melliturga*) and used to map the ancestral hosts. Exceptions to this practice were the most frequent hosts of the genera *Lasioglossum* and *Halictus*, which were each divided into two main lineages according to their phylogeny (*Lasioglossum* sensu stricto and other *Lasioglossum* lineages and *Halictus* subgenus *Halictus* and *Halictus* subgenus *Seladonia* lineages) [58].

#### Ancestral host specificity

We classified all *Sphecodes* species as either specialists or generalists according to their known host range(s) [55], [56], [57], [39]. However, there are no agreed-upon definitions of specialists and generalists, and moreover, the two categories are continuous rather than discrete. Therefore, the fundamental question is the position of the dividing line between the specialists and the generalists. Several criteria can be used to estimate the degree of resource specialization. The most commonly used criterion is the number of resources utilized by a species. Other criteria that can be considered are the differences in the intensity of use of particular resources [59], the degree of phylogenetic distinction between the utilized resources [60], the ecological distinction between the utilized resources and the number of adaptations necessary for successful resource use. There are several indices available that measure the degree of host specificity in parasites [61]; however, such indices require detailed knowledge concerning the individual species, which is not available for *Sphecodes*. Moreover, none of these indices account for ecological differences between the utilized resources. Therefore, we did not rely on any existing index; instead, we defined specialists and generalists as a function of the phylogenetic and ecological distinctions between the utilized resources. To validate our approach, we used the following three alternative definitions and thus three alternative distributions of specialists and generalists.

#### Distribution I – the most unrestrictive definition of specialists

1) Specialists comprise species whose hosts represent a single genus or include one additional host of a different genus, but the second host is only sporadically used. 2) Generalists include species that do not meet either of the above criteria for specialists.

#### Distribution II – used for our main analysis

1) Specialists comprise species with ecologically similar hosts of the same genus or with one additional host of a different genus that is nonetheless ecologically similar to the other hosts and only sporadically utilized. 2) Generalist species do not meet either of the above criteria for specialists.

#### Distribution III – the most restrictive definition of specialists

1) Specialists comprise species with ecologically similar and related hosts that represent a single genus. 2) Generalist species do not meet this criterion for specialists.

### Reconstruction of ancestral states

Each terminal in the *Sphecodes* phylogenetic tree was coded by character state, that is, either by host type (*Lasioglossum* s. s., other *Lasioglossum*, *Halictus* subgenus *Seladonia*, *Halictus* subgenus *Halictus*, *Andrena*, *Colletes*, *Perdita* and *Melliturga*) for analyses of the reconstruction of the ancestral host group or by behavioral category (specialist and generalist) for analyses of the reconstruction of ancestral host specificity. For the species with no known host, the terminal was coded as “missing data”. Two methods for reconstructing the ancestral states were used: the Bayesian method using the program BayesTraits [62] and the maximum parsimony method using the program Mesquite [63].

#### Bayesian method

A tree file was created from the Bayesian analysis of the phylogeny. Each 10,000th tree (each 10th saved tree) of each independent run was selected except for the first 25% of trees, which were discarded as burn-in. Therefore, the complete tree file consisted of 1,500 trees. Three alternative data files (according to the three alternative distributions of specialists and generalists) for the reconstruction of ancestral host specificity were created. Ancestral states for all the nodes were calculated for Distribution II. Only the ancestral states of Node 1 (i.e., the most recent common ancestor of the genus *Sphecodes*) were calculated for Distributions I and III.

An independent analysis for each host type was performed in the mapping of the ancestral hosts. Thus, a data file for each host was created with both the presence and the absence of the host in each *Sphecodes* species. We chose this method instead of using only one analysis with multiple character states to assign independent posterior probabilities for several different hosts at one ancestral node. This model reflects independent transitions between the host genera in cuckoo bee evolution, unlike the standard multiple-character analysis in which the posterior probability is distributed within all the states and provides an inconclusive result concerning the number of possible host genera at the ancestral nodes. Multiple-character analysis of all the genera together can well resolve which host genus was more probable at which ancestral node, but this is not the question here. The use of the generic level for hosts, however, underestimates the host switches but better reflects the current knowledge of the host associations included in the model. Studied host switches quite likely also exist on the subgeneric level, but these switches are missed in our study.

We initially performed the ML analyses for each set of characters (hosts, Distribution I, Distribution II and Distribution III). These analyses computed the optimal transition rates from one character state to another for each tree in the tree file. The settings of the Bayesian analyses were subsequently optimized to best reflect the distribution of the transition rates obtained from the ML analyses.

A reversible jump model with a hyperprior was used for all the analyses. The reversible jump model searches among the possible models of trait evolution (those with the same versus different transition rates between character states) and visits these models in proportion to their posterior probabilities [64]. Hyperprior means that the program estimates priors from the data using a uniform hyperprior to seed the priors [62].

The priors were obtained from an exponential distribution in the interval 0–20 for the reconstruction of the ancestral host group, 50–200 for the reconstruction of the ancestral host specificity using Distributions II and III, or 0–70 for the reconstruction of ancestral host specificity using Distribution I.

The ratedev parameter, which specifies the magnitude of change proposed to rate the coefficients at each iteration of the chain, was optimized for the resulting acceptance rate of the newly proposed values of the rate parameters between 20–40%, as is recommended in the manual [62].

The analysis was run for either 5 million (the reconstruction of ancestral hosts) or 50 million (the reconstruction of ancestral host specificity) generations. The estimated parameters were checked for convergence using Tracer 1.4 [52]. The first 2 million (the reconstruction of ancestral host) or 40 million (the reconstruction of ancestral host specificity) generations were discarded as burn-in.

The mean of the posterior probabilities of the character state was calculated for each node. Only hosts with a posterior probability greater than 0.7 were considered as possible hosts.

To test whether there is significant support for a character state with a higher posterior probability, an MCMC analysis with a fixed character state as a specialist and alternatively as a generalist was performed for 50 million generations for each node. The harmonic means resolved from these two analyses were compared using the Bayes factor (see above). Values above 2 are considered to indicate positive support, those above 6 are considered to indicate strong support, and those above 10 are considered extremely strong support [50].

#### Maximum parsimony method

A consensus tree generated from the Bayesian phylogenetic analysis was used for the maximum parsimony ancestral mapping method. The ancestral host reconstruction and the three alternative analyses of ancestral host specificity reconstruction corresponding to the three alternative distributions of specialists and generalists were performed using the program Mesquite [63].

### Test of irreversible evolution

We tested whether evolution is directional, i.e., whether specialists can arise from generalists, but generalists cannot arise from specialists (or vice versa), or flexible, i.e., specialists can arise from generalists, and vice versa. Therefore, a model allowing transitions in both directions was compared to a model allowing transitions in only one direction. Likelihoods for each tree in the tree file (the same tree file as that used in the character mapping analyses) were calculated using the program BayesTraits for (i) a model allowing transitions in both directions, (ii) a model allowing transitions only from generalist to specialist and (iii) a model allowing transitions only from specialist to generalist. The mean of the likelihoods was calculated for each model, and the model means were compared using the likelihood ratio test. The two types of model differ by one free parameter, and the resulting value approximates a chi-square distribution with one degree of freedom [62].

## Results

### Phylogeny of subtribe Sphecodina

Both phylogenetic analyses (Align1 and Align2) were performed without dataset partitioning. We applied the DNA substitution model, GTR + Γ + I, to the entire dataset. This partitioning strategy is suitable for the ML method (for Align1, AIC [no partitioning]  = 64,992, AIC [best partitioning schema for each gene]  = 65,048, and AIC [the most partitioned schema]  = 65,078; for Align2, AIC [no partitioning]  = 53,240, AIC [best partitioning schema for each gene]  = 53,246, and AIC [the most partitioned schema]  = 53,260) as well as for the Bayesian method, where all the comparisons of 2 ln (BF) between models were <2.

**Figure 1:**
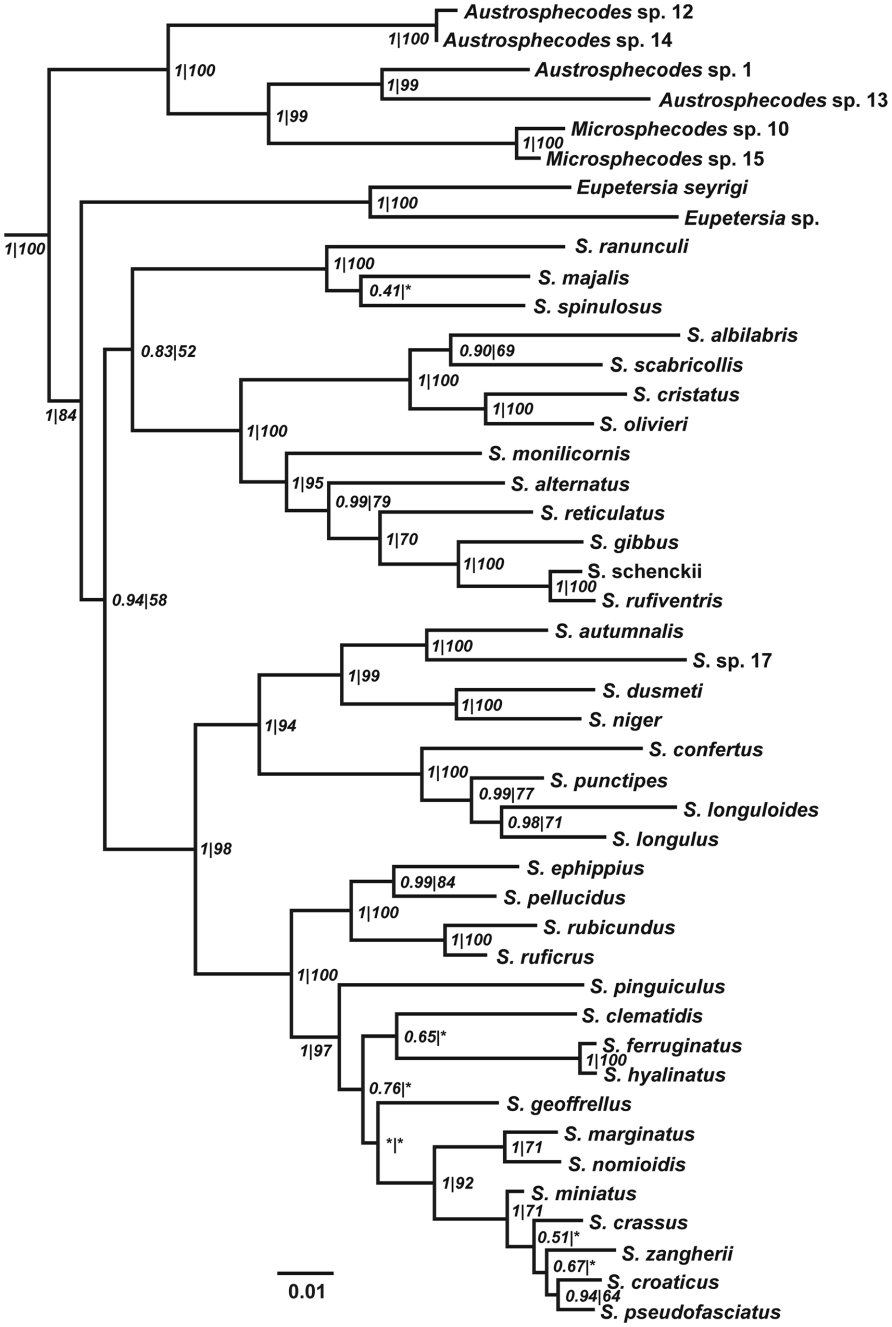
Phylogenetic tree resulting from Bayesian analysis of Align1. Posterior probabilities are in front of the slash and bootstrap values from the maximum likelihood analysis are behind the slash. Posterior probabilities below 0.5 and bootstrap values below 50 are replaced by an asterisk (*).

All the phylogenetic analyses (the Bayesian and ML methods for Align1 and Align2) resulted in robust phylogenies, all of which were congruent at the majority of nodes (Figs. 1 and S1, all trees are available from www.treebase.org). There is some difference between the tree topologies constructed from the five- and the three-gene analyses; however, branch support increases with the addition of sequential information at the majority of nodes. The main difference between the resulting trees concerns the ambiguity of branching of the basal lineages of the subtribe Sphecodina. Both of the analytical methods used for branch support (posterior probability in the Bayesian and bootstrap in the ML analysis) give good support at most nodes for Align1 (Figs. 1 and S1). The Bayesian tree with topologies based on the analysis of Align2 with posterior probabilities and the ML bootstrap support are available in the Supporting Information (Fig. S1).

Two taxa used in our study from the *Microsphecodes* genus cluster within four specimens of A*ustrosphecodes*, which renders *Austrosphecodes* a strongly supported paraphyletic group (Fig. 1).

The consensus tree from the Bayesian analysis, along with the ML tree, suggests the monophyly of *Sphecodes* but with weak statistical support (Align1 posterior probability 0.94, bootstrap 58; Align2 posterior probability 0.83, bootstrap 51). It is unclear whether the genus *Eupetersia* forms a sister group of genus *Sphecodes* or belongs within it.

The position of the clade consisting of *S. majalis*, *S. spinulosus* and *S. ranunculi* (*Proteraner* group) is uncertain because its support within the genus *Sphecodes* was very weak (Align1 posterior probability 0.83, bootstrap 52; Align2 posterior probability 0.53) and even absent based on the ML analysis of Align2 in which this *Proteraner* group appears as a sister group to all the other *Sphecodes* species. Some other *Sphecodes* species were problematic in our analysis, e.g., *S. pinguiculus*, *S. geoffrellus* and the entire *S. marginatus* group (Figs. 1 and S1).

All of the specimens of the morphologically and behaviorally diversified species known to be individual specialists (*S. ephippius* and *S. monilicornis*) are well supported as members of a monophyletic group and do not vary more than specialized species in branch lengths (Fig. S1).

### Ancestral host group

**Figure 2:**
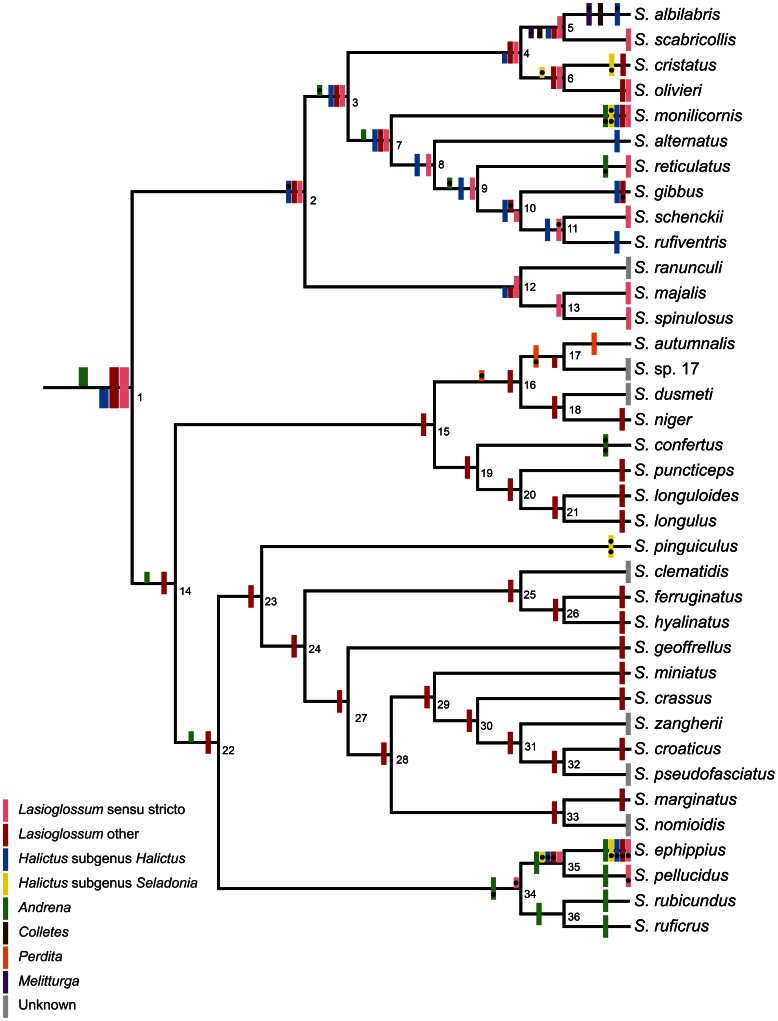
Character state reconstruction of ancestral hosts of the cuckoo bees of the genus ***Sphecodes*** **.** The possible hosts identified by Bayesian analysis (posterior probability >0.7) are shown above the nodes and those identified by maximum parsimony analysis are shown below the nodes. Black spots indicate host switches. Specific values of the posterior probabilities are shown in Table S2.

Both of the ancestral mapping methods (Bayesian and maximum parsimony) identified the ancestral *Sphecodes* s.l. as having three host lineages: *Lasioglossum* s. s., other *Lasioglossum* and either *Andrena* (according to the Bayesian method) or *Halictus* (according to the maximum parsimony method) (Fig. 2, Table S2). Both analyses showed that switches between hosts have commonly occurred (Fig. 2). These switches have occurred 17 times at 13 ancestral nodes of *Sphecode* according to the Bayesian analysis and 16 times at 10 ancestral nodes according to the maximum parsimony analysis (Fig. 2). Only three recognized switches were the same in both analyses: *S. monilicornis* (switching to *Halictus* subgenus *Seladonia* within its five host groups), *S. confertus* and *S. pinguiculus*. In ten cases, it is clear that the switches correspond to one another, although they occur at different locations in each analysis (the localization of the host group according to the Bayesian analysis precedes the dash): Node 5– *S. albilabris* (switches to *Colletes* and *Melitturga*), Node 6– *S. cristatus* (switch to *Halictus* subgenus *Seladonia*), Node 9– *S. reticulatus* (switch to *Andrena*), Node 10– *S. gibbus* (switch to *Lasioglossum*), Node 16– Node 17 (switch to *Perdita*), Node 34– *S. ephippius* and *S. pellucidus* (switch to *Lasioglossum* s. s.), and Node 35– *S. ephippius* (switch to *Lasioglossum* other, *Halictus* subgenera *Halictus* and *Seladonia*). In four cases, a switch is apparent from the Bayesian analysis but not the maximum parsimony analysis: Node 2 (switch to *Halictus*), Node 3 (switch to *Andrena*), *S. albilabris* (switch to *Halictus*) and Node 11 (switch to *Lasioglossum* s. s.). In one case, a switch apparent from the maximum parsimony analysis is absent from the results of the Bayesian analysis: Node 34 (switch to *Andrena*). A complete list of the posterior probabilities of the particular ancestral states is given in Table S2.

### Evolution of host specificity

We performed host specificity analyses using three alternative definitions of specialist and generalist, which have different character distributions in the phylogeny of *Sphecodes*. Based on our definitions of host specificity, we identified 2, 8 and 9 host generalists (using Distributions I, II and III, respectively) from 30 species with known host records and 6 distinct species with unknown host records. Other *Sphecodes* specimens belonging to taxa with unknown host records as well as unidentified specimens were not included in the analysis. According to the likelihood ratio test and using Distribution II or III, the bidirectional model for the evolution of host specificity (character states can change in either direction between specialist and generalist) has a significantly higher likelihood than any unidirectional model. The likelihood ratio test using Distribution I indicates that the bidirectional model is significantly better than the unidirectional model for change from generalist to specialist, but the unidirectional model for change from specialist to generalist is not significantly worse than the bidirectional model (Table 4).

**Table 4:** Results of likelihood ratio test.

Distribution I			
	G ↔ S	G → S	S → G
**Mean of ln likelihood**	−6.545	−8.731	−7.850
**Result of LR test**		4.370	2.610
**P** – **value**		**0.0365**	**0.1061**

Distribution II			
	G ↔ S	G → S	S → G
**Mean of ln likelihood**	−16.592	−20.418	−20.119
**Result of LR test**		7.652	7.054
**P** – **value**		**0.0056**	**0.0079**

Distribution III			
	G ↔ S	G → S	S → G
**Mean of ln likelihood**	−17.494	−21.399	−21.581
**Result of LR test**		7.810	8.173
**P** – **value**		**0.0051**	**0.0042**

Comparison of results from one-direction models of character evolution with an unrestricted model for specialists (S) and generalists (G) for three definition criteria of host specialization (Distributions I-III).

The results of the host specificity mapping are largely ambiguous. According to the Bayesian ancestral state reconstructions, the specialist state is the more probable host specificity at the majority of nodes, including the most recent common ancestor of *Sphecodes* (Table S1, Fig. S2). However, these results are mostly inconclusive. The Bayes factor positively supports particular ancestral states only in 4 cases (specialists at nodes 11, 15, 26, and 36) (Table S1, Fig. S2). The BF value is less than 2 (indicating no support) for the remaining nodes. These results indicate that generalists and specialists are approximately equally probable for the majority of nodes, and we cannot determine whether the ancestors of certain extant species were specialists or generalists.

According to the maximum parsimony analyses, the generalist state occurs only at Node 35 using Distribution III. Both states are recognized as possible at Nodes 7, 9, and 10 using Distribution II and at Nodes 3, 4, 5, 6, 7, 9, and 10 using Distribution III. For the remaining nodes, the specialist state is the more probable (Fig. S2). According to the results of the maximum parsimony method, the most recent common ancestor of *Sphecodes* was a specialist, and generalist species must have arisen from specialist ancestors only recently.

## Discussion

### Phylogeny and systematics

Our results support the monophyly of the subtribe Sphecodina in accordance with previous assumptions [40], [24] but conflict with other suggested relationships within this subtribe (Fig. 1, see also Fig. S1). Michener [40], [24] recognizes *Austrosphecodes* as a subgenus of *Sphecodes* and *Microsphecodes* as a separate genus from *Sphecodes*. However, our results do not support this classificatory hypothesis. Our analyses suggest that *Austrosphecodes* is not closely related to *Sphecodes* but is a basal lineage of Sphecodina, and *Microsphecodes* is an inner group of *Austrosphecodes*, thus rendering the latter paraphyletic (Fig. 1). *Ptiolocleptis* (missing in our study) may also be a representative of this lineage [40]. Little is known about the diversity and species relationships within this Neotropical lineage, and thus, other taxonomic suggestions would not currently be useful (e.g., dividing all lineages into different genera or subgenera). The genera *Eupetersia* and *Sphecodes* are recognized as monophyletic by Michener [24]; however, clade support for this supposition was relatively weak in our analysis, although we omit certain taxa that may affect inference of the basal relationships of this subtribe (*Callosphecodes* Friese and *Nesosphecodes* Engel) [65], [66]. Further studies are necessary to resolve the basal relationships within the subtribe Sphecodina and the systematics of *Sphecodes* at the subgeneric level. The groups established by Robertson [67] based on morphological descriptions, such as *Proteraner* (here, *S. majalis*, *S. ranunculi* and *S. spinulosus*) or *Drepanium* (here, *S. confertus*, *S. longuloides*, *S. longulus* and *S. punctipes*) seem plausible. Our phylogenetic analysis and the preexisting keys (based on morphology [39]) sort species into the same groups across studies, which may enable researchers to understand characters in this problematic diversified group and permit the further distinction of *Sphecodes* species to the level of subgenera. Such taxonomic studies will enhance detailed alpha-taxonomic, morphological and behavioral studies in the future.

### Evolution of host specificity

*Sphecodes* cuckoo bees appear to be largely specialists regarding host choices. Only two of the thirty analyzed species are obvious generalists, although an additional seven exhibit host variability [39]. We mapped host specialization strategies and reconstructed ancestral strategies in *Sphecodes* cuckoo bees using two different methods (Bayesian and maximum parsimony), but the results of the two methods are incongruent. The results of the Bayesian analysis are largely inconclusive; therefore, the likelihood ratio test of the host specialization evolutionary model seems to be the more relevant approach for answering the question about evolution of host specialization. The likelihood ratio test using Distribution II and III strongly supports the occurrence of clear transitions between generalists and specialists in both directions in the evolution of the genus *Sphecodes*. Unidirectional evolution from generalists to specialists or from specialists to generalists is significantly rejected. The results of the likelihood ratio test using Distribution I are not so convincing, but the transition from generalists to specialists is also significantly rejected (Table 4). Although the generalist state is traditionally considered to be the ancestral state and to later lead to the derived condition (specialization) [7], here we provide evidence of both types of evolutionary change. There are two possible reasons for the reversal to the ancestral state and back.

First, perhaps the generalist state is not the ancestral one. According to Emery [68], social ant parasites arise from their hosts as a result of sympatric speciation and must therefore be specialists at the outset. Nevertheless, environmental changes apply selective pressures to all parasitic species, and sometimes, adaptation to this situation requires the adoption of new or additional host species to avoid extinction. If such host switches are successful, parasitic species may become generalists (at least for a short time). Several authors adopted this theory, recently interpreted as Emery's rule, e.g., Carpenter et al. [69], who applied the theory to explain the specialization of cuckoo wasps to their closely related hosts. Emery's rule has been used for social parasites, but we do not know if it applies to nest cleptoparasites.

Second, *Sphecodes* cuckoo bees may not have reverted to the generalist strategy as understood for nonspecialized ancestors, but may have adopted a new generalist strategy of specialization at the individual level. This strategy occurs in two European *Sphecodes* species (*S. monilicornis* and *S. ephippius*), which have the broadest host range [38]. Specialization at the individual level allows species to take advantage of a large number of hosts as well as potentially adapt to a particular host or at least overcome certain constraints. Such specialization might be coded as a third type of host strategy, along with specialist and generalist.

Third, generalist species can consist of distinct genetically separated lineages, each specialized to a different host. However, no clustering within lineages is evident for *S. ephippius* or *S. monilicornis* in our phylogenetic trees based on all of the samples (Fig. S1), albeit that the genes chosen for our study could be inappropriate for resolution of relationships among such close lineages.

Our results based on the mapping of host specificity and the likelihood ratio test are likely biased by the problematic determination of the basic distinction between the specialist and generalist strategies. There is also the limitation that the mapping of ancestral host specificity cannot detect a host switch that occurs without a change in host specificity strategy. For these reasons, the character state reconstruction of the ancestral host group of *Sphecodes* cuckoo bees offers a better approach and, unlike the mapping of host specificity, provided comparable results using both applied methods (the Bayesian and maximum parsimony analyses). Practically, this reconstruction provides a different approach to the same problem discussed above and can also determine the number of possible host switches and the host lineages that become new hosts. Despite obvious limits on lineage resolution, we can identify the most prominent host changes. There are between 11 and 13 clades with 16 to 17 detected host changes (depending on analysis method) in the evolutionary history of the analyzed *Sphecodes* species (Fig. 2). Although we missed all the host switches below the resolution of our host definitions, our results suggest that cuckoo bees are relatively flexible in their host choice. Thus, there is a high probability that our finding of multiple switches between the generalist and specialist strategies in cuckoo bee ancestors is valid.

We obtain a similar result from the analysis of the most recent common ancestor of all the *Sphecodes* species. It is difficult to infer whether this ancestor was a specialist or a generalist, but it seems to have been a parasite of two or possibly three different lineages (Figs. 2 and S2). However, a pitfall of the ancestral host group reconstruction method arises because some identified hosts might not have yet existed when the ancestral *Sphecodes* species lived and diverged. According to Gibbs et al. [58], [70], the most recent common ancestor of *Sphecodes* lived before approximately 25 MYA, and the divergence of host *Lasioglossum* lineages (*Lasioglossum* s. s. and other subgenera of this genus) occurred before approximately 30 MYA. These results suggest that the *Lasioglossum* lineages had already diverged when the MRCA of *Sphecodes* arose. However, the credible dating intervals are quite large and overlap each other (18–4 MYA for the MRCA of *Sphecodes* and 25–7 MYA for divergence of the *Lasioglossum* lineages). Therefore, cospeciation of the *Sphecodes* ancestor with the *Lasioglossum* ancestor is also a possible scenario.

Although *Lasioglossum* is strongly supported as an ancestral host lineage, hosts from *Andrena* or the *Halictus* lineage are also likely for the most recent common ancestor of *Sphecodes*. These host groups were already established when the ancestral *Sphecodes* species lived and diverged [71].

### Host switches and constraints for the generalist strategy

According to our results, specialization is not an evolutionary dead end in cuckoo bees of the genus *Sphecodes*, even though the majority of extant species are specialists (Figs. 2 and S2). Our results clearly suggest that the specialists do not depend entirely on their hosts but likely have a range of potential host species (or lineages) that might not be optimal but are sufficient for survival. According to Agosta and Klemens [22], such a potential host pool may provide a sloppy fitness space (i.e., a potential fitness space outside the range of conditions in which the species evolved). Such a fitness space may enable a switch to another host species. Nevertheless, we could obtain the same result if a large number of extinct species existed and they were all strictly dependent on their hosts (i.e., unable to switch to alternate hosts). Knowledge of the extinction rate of these cuckoo bees would be informative here; however, no fossil cuckoo bees are known (M. S. Engel, personal comm.). We prefer the former hypothesis because the behavior of some recent *Sphecodes* species suggests the successful utilization of different hosts following exposure to ecological pressure. For example, *S. albilabris* uses the early spring bee, *Colletes cunicularius*, as the primary and most likely essential host [55], [72], but this parasite lives longer than its spring host and utilizes various species similar in size to *C. cunicularius* during the summer [39]. These hosts (e.g., *Meliturga clavicornis* [73] and *Halictus quadricinctus* [39]) are phylogenetically unrelated to the main host species and to each other. As our results show, cuckoo bees are most likely not obligately fixed to their hosts; therefore, host switches are more likely than cospeciation events. Nevertheless, there must be certain constraints that inhibit the arbitrary switching of hosts. If so, the most advantageous strategy would be generalism. Three constraints that may act synergistically are the most probable:

1. Pollen specialization of host species. Pollen-collecting bee species are physiologically constrained in their choice of flowers due to pollen toxicity [25], [26], and it is likely that the host choices of cuckoo bees are physiologically constrained as well. Appropriate hosts are only those that provide pollen appropriate for the growth of the immature cuckoo stages.
2. Neurological constraints. Bees have limited memory and learning capacities [28], [4]. The limitations of parasitic bees may be similar to the constraints involved in flower constancy [32]. There are likely several different factors involved in finding and using an appropriate host, requiring various olfactory, visual and strategic abilities. The brains of bees can concentrate only on a single target at a time and can be effective only in such cases.
3. Size ratio of a cuckoo bee to its host. A host smaller than the size of a cuckoo bee would likely be unsuitable. First, the amount of pollen provided may be insufficient for the development of a larva of a large cuckoo bee. This constraint may not be overwhelming because there is considerable variability in the size of generalists (*S. monilicornis* as well as *S. ephippius*) (Bogusch and Straka, personal observation). A more important consideration may be the nest entrance size because adult females must be able to enter the host nests. Adult female *S. crassus* that had most likely grown up in cells designated for queens of the social species, *Lasioglossum pauxillum*, were unable to penetrate the nests of the same host species after reduction of the entrance size to fit smaller workers of *L. pauxillum* (J. Straka, personal observation).

Cuckoo bees face one or more of these constraints when switching to ecologically dissimilar hosts. However, these constraining factors need not be limiting if the switch occurs within ecologically similar hosts. Therefore, there is a large difference between a “true generalist” with many ecologically dissimilar and unrelated hosts and a “faux generalist”, which is specialized on only a phylogenetically diversified lineage of hosts [74]. Moreover, there could also be “faux specialists”, which are able to utilize more ecologically dissimilar hosts but remain restricted only to a few ecologically similar resources while the others are hidden (for example due to competition or nonoverlapping distributional ranges) [74]. Therefore, the host specificity of a particular species should be determined by its ability to overcome the above-mentioned constraints. Other methods do not permit the detection of false specialists and generalists.

True generalist species often use dissimilar hosts from various families [38]. How do they overcome these constraints? One possibility is specialization at the individual level, which reduces intraspecific competition and enhances host utilization by individuals. Such generalist species can accept almost any host, even when they are eusocial. Both *S. ephippius* and *S. monilicornis* invade the nests of both solitary and eusocial bee species [39].

We can also mention that there are many pollen-collecting bee species that are not attacked by *Sphecodes*, some that are even closely related to the host species. Such species might be inappropriate for the parasite for several reasons different from the already mentioned constraints. These species might be shifted in time, biotope or biogeography and do not meet the parasite; they might have an effective protective behavior against the parasite evolved during past contact with *Sphecodes*; or they might display protective behavior against another extant cuckoo parasite from a different lineage, which protects them from effective utilization by *Sphecodes*. The latter behavior can involve nest construction habits, such as closure of the nest entrance or the digging of a fake nest entrance [75].

### Summary of observed behavioral patterns and individual choice constancy

We can find several different life strategies in *Sphecodes* cuckoo bees. Specialization at the individual level such as that currently known in two unrelated generalist species (*S*. *monilicornis* and *S*. *ephippius*) seems to occur only rarely. Other species are usually connected to a single host species (or genus) at one locality and time. Nevertheless, at different localities or times, one can find the same specialized species or even the same individuals parasitizing different hosts unrelated to the previously known hosts. Alternative hosts are well described in *Sphecodes albilabris* [39], [73], and such parasitizing flexibility may also be true for *S. cristatus*, *S. olivieri*, *S. reticulatus*, *S. geoffrellus*, *S. crassus*, *S. pellucidus*, and certain other species that are expected to be specialists but also have different unexpected hosts proposed for them [39]. So-called generalists that are actually specialists at the individual level solved the problem of how to exploit a potentially rich host pool, but other species, even the specialized ones may be able to switch hosts under certain circumstances. Given our results, we can suggest that a flexible host utilization approach should be widespread or even general for cuckoo bees, and thus, the host utilization strategy should be interpreted more broadly than simple generalist and specialist. We have shown that this differentiation into specialists and generalists hardly works at all. Therefore, we looked for analogous patterns of flexible host utilization and for frequent host switches in the evolution of bees, and we found similarities between the choice of host in cuckoo bees and the choice of flower in pollen-collecting bees (flower constancy) [32], [38]. We can imagine host choice as a specialization at the individual level in all species, with individual variability in the decision strategy to switch hosts. Of course, individuals of some species are more constant in their host choice and change the host less frequently (specialists) than others (generalists). However, the degree of flexibility in the host choice of species (as well as individuals) falls more on a continuum than in discrete categories (specialist and generalist). Moreover, various ecological pressures could limit the availability of the preferred host at a given time and therefore the willingness of a single individual (as well as the whole population sharing the same constraints) to switch hosts can vary with time. For example, when an individual of a specialized species has many available oocytes remaining in its ovaries and is unsuccessful in finding an appropriate host, the decision to switch hosts may be made more readily and thus quite frequently. This decision pattern derives from the flower constancy example and can also likely be applied to other choices by bees. The pattern may well represent a common behavioral strategy for decision making to overcome various constraints in bees and other Hymenoptera; we refer to the pattern as “individual choice constancy”. This hypothesis must be tested in the future, but observations from *Sphecodes* bees are consistent with this view, including the results of laboratory studies, particularly those performed with *S. pellucidus* [76]. This is the only well-documented case of host switching (from *Andrena* host to *Lasioglossum*) in a potentially specialist species [39]. Conducting similar, more detailed experiments with choice pressures in a highly specialized species would be informative. There are also interesting field observations concerning the existence of host “races” in cuckoo bees that differ in size, e.g., in the genus *Coelioxys* [77]. These observations also suggest that individual specialization is modulated by the constraints proposed in this paper. Experiments varying the size of the cuckoo bee, the pollen source and the configuration of host nests and their entrances would be useful for understanding the existence and evolutionary importance of individual choice constancy in cuckoo bees.

## Supporting Information

Figure S1 **Phylogenetic tree resulting from Bayesian analysis of a complete dataset (Align2).** Posterior probabilities are in front of the slash; bootstrap values of the maximum likelihood analysis are behind the slash. Posterior probabilities lower than 0.5 and bootstrap values lower than 50 are replaced by an asterisk (*).
(TIF)

Figure S2 **Character state reconstruction of host specificity in cuckoo bees of the genus** ***Sphecodes*** **.** The larger pie charts show posterior probabilities obtained by Bayesian analysis using Distribution II (main analysis). The smaller pie charts show the results (posterior probabilities in Bayesian analyses or possible character states in maximum parsimony analyses) of other analyses in which interpretations differed from those of the main analysis. B1, Bayesian analysis using Distribution I; MP1, maximum parsimony analysis using Distribution I; B3, Bayesian analysis using Distribution III; and MP3, maximum parsimony analysis using Distribution III.
(TIF)

Table S1 **Complete results of character state reconstruction of host specifity.** DI: Distribution of specialist and generalist I, DIII: Distribution of specialist and generalist III, P(G): posterior probability for generalism as ancestral state, P(S): posterior probability for specialism as ancestral state, BF: Bayes Factor (support of more probable state). Values of BF lower than 2 are replaced by asterisk (*).
(DOC)

Table S2 **Complete results of character state reconstruction of ancestral hosts.** P (LS): posterior probability for *Lasioglossum* s. s. as ancestral host, P (LO): posterior probability for *Lasioglossum* other as ancestral host, P (H): posterior probability for *Halictus* subgenus *Halictus* as ancestral host, P (S): posterior probability for *Halictus* subgenus *Seladonia* as ancestral host, P (A) posterior probability for *Andrena* as ancestral host, P (C): posterior probability for *Colletes* as ancestral host, P (P): posterior probability for *Perdita* as ancestral host, P (M): posterior probability for *Melitturga* as ancestral host. The values higher than 0.7 are in **bold**.
(DOC)
