# Supplementary figures and images for: Flexible Host Choice and Common Host Switches in the Evolution of Generalist and Specialist Cuckoo Bees (Anthophila: Sphecodes)
Source: PLoS One. 2013 May 17;8(5):e64537. doi: 10.1371/journal.pone.0064537 (PMC3656848; doi:10.1371/journal.pone.0064537)

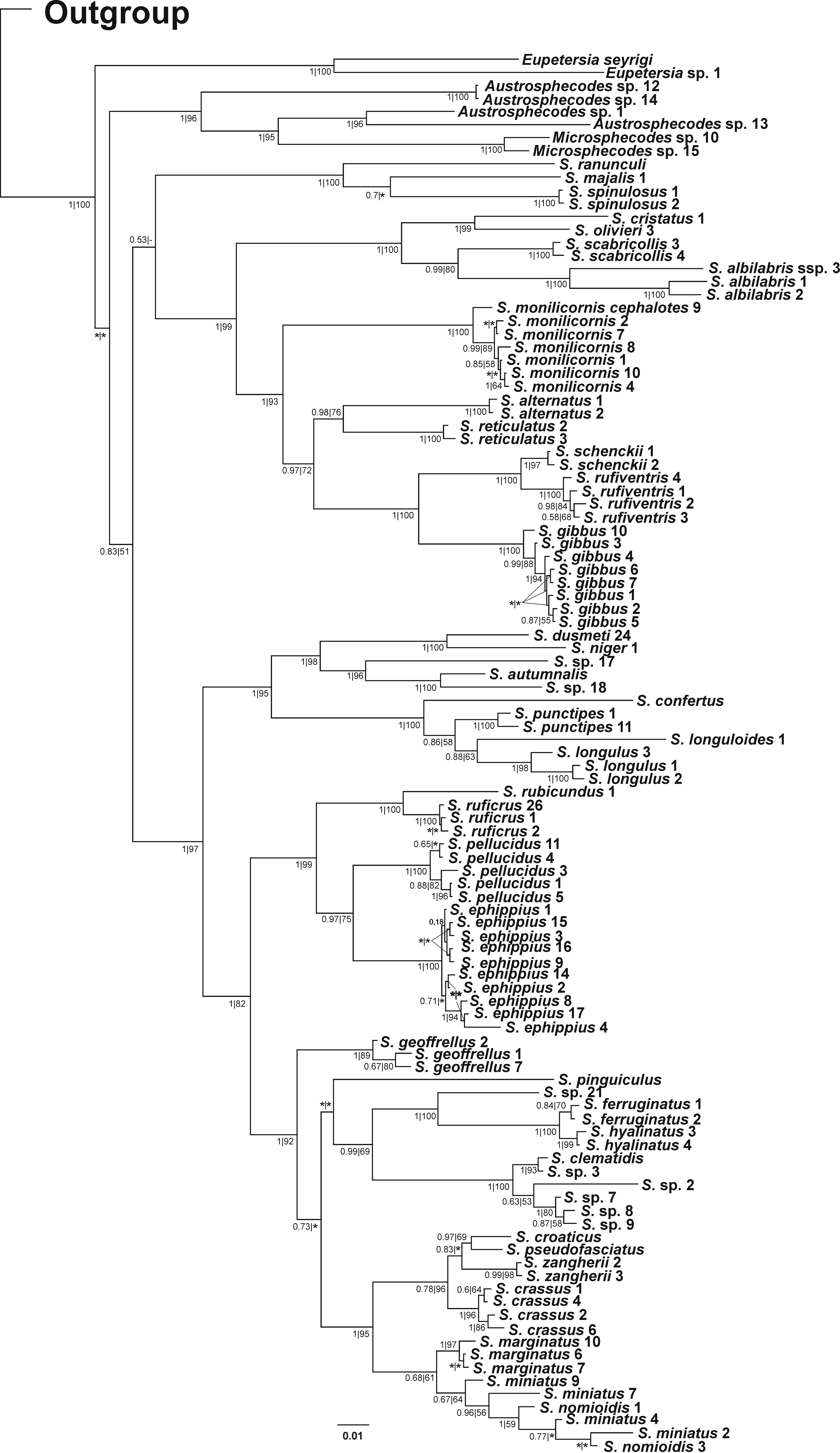

Supplement: Figure S1 — Phylogenetic tree resulting from Bayesian analysis of a complete dataset (Align2). Posterior probabilities are in front of the slash; bootstrap values of the maximum likelihood analysis are behind the slash. Posterior probabilities lower than 0.5 and bootstrap values lower than 50 are replaced by an asterisk (*). (TIF) [file pone.0064537.s001.tif]
