# Supplementary material for: Flexible Host Choice and Common Host Switches in the Evolution of Generalist and Specialist Cuckoo Bees (Anthophila: Sphecodes)
Source: PLoS One. 2013 May 17;8(5):e64537. doi: 10.1371/journal.pone.0064537 (PMC3656848; doi:10.1371/journal.pone.0064537)
Supplement: Table S1 — Complete results of character state reconstruction of host specifity. DI: Distribution of specialist and generalist I, DIII: Distribution of specialist and generalist III, P(G): posterior probability for generalism as ancestral state, P(S): posterior probability for specialism as ancestral state, BF: Bayes Factor (support of more probable state). Values of BF lower than 2 are replaced by asterisk (*). (DOC) [file pone.0064537.s003.doc]

|  | **P (G)** | **P (S)** | **BF** |
| --- | --- | --- | --- |
| **Node1 DI** | 0.37 | 0.63 | * |
| **Node1** | 0.45 | 0.55 | * |
| **Node1 DIII** | 0.48 | 0.52 | * |
| **Node2** | 0.47 | 0.53 | * |
| **Node3** | 0.51 | 0.49 | * |
| **Node4** | 0.42 | 0.58 | * |
| **Node5** | 0.48 | 0.52 | * |
| **Node6** | 0.33 | 0.67 | * |
| **Node7** | 0.55 | 0.45 | * |
| **Node8** | 0.50 | 0.50 | * |
| **Node9** | 0.57 | 0.43 | * |
| **Node10** | 0.49 | 0.51 | * |
| **Node11** | 0.17 | 0.83 | 2.14 |
| **Node12** | 0.37 | 0.63 | * |
| **Node13** | 0.37 | 0.63 | * |
| **Node14** | 0.44 | 0.56 | * |
| **Node15** | 0.39 | 0.61 | 2.43 |
| **Node16** | 0.39 | 0.61 | * |
| **Node17** | 0.38 | 0.62 | * |
| **Node18** | 0.39 | 0.61 | * |
| **Node19** | 0.34 | 0.66 | * |
| **Node20** | 0.29 | 0.71 | * |
| **Node21** | 0.33 | 0.67 | * |
| **Node22** | 0.47 | 0.53 | * |
| **Node23** | 0.44 | 0.56 | * |
| **Node24** | 0.45 | 0.55 | * |
| **Node25** | 0.43 | 0.57 | * |
| **Node26** | 0.10 | 0.90 | 10.83 |
| **Node27** | 0.48 | 0.52 | * |
| **Node28** | 0.37 | 0.63 | * |
| **Node29** | 0.31 | 0.69 | * |
| **Node30** | 0.45 | 0.55 | * |
| **Node31** | 0.29 | 0.71 | * |
| **Node32** | 0.29 | 0.71 | * |
| **Node33** | 0.30 | 0.70 | * |
| **Node34** | 0.54 | 0.46 | * |
| **Node35** | 0.69 | 0.31 | * |
| **Node36** | 0.24 | 0.76 | 2.36 |
