# Supplementary material for: Flexible Host Choice and Common Host Switches in the Evolution of Generalist and Specialist Cuckoo Bees (Anthophila: Sphecodes)
Source: PLoS One. 2013 May 17;8(5):e64537. doi: 10.1371/journal.pone.0064537 (PMC3656848; doi:10.1371/journal.pone.0064537)
Supplement: Table S2 — Complete results of character state reconstruction of ancestral hosts. P (LS): posterior probability for Lasioglossum s. s. as ancestral host, P (LO): posterior probability for Lasioglossum other as ancestral host, P (H): posterior probability for Halictus subgenus Halictus as ancestral host, P (S): posterior probability for Halictus subgenus Seladonia as ancestral host, P (A) posterior probability for Andrena as ancestral host, P (C): posterior probability for Colletes as ancestral host, P (P): posterior probability for Perdita as ancestral host, P (M): posterior probability for Melitturga as ancestral host. The values higher than 0.7 are in bold. (DOC) [file pone.0064537.s004.doc]

|  | **P (LS)** | **P (LO)** | **P (H)** | **P(S)** | **P (A)** | **P (C)** | **P (P)** | **P(M)** |
| --- | --- | --- | --- | --- | --- | --- | --- | --- |
| **Node1** | **0.95** | **0.96** | 0.64 | 0.33 | **0.71** | 0.12 | 0.16 | 0.12 |
| **Node2** | **0.98** | **0.79** | **0.80** | 0.44 | 0.61 | 0.20 | 0.18 | 0.20 |
| **Node3** | **0.98** | **0.90** | **0.95** | 0.60 | **0.75** | 0.18 | 0.11 | 0.18 |
| **Node4** | **0.94** | **0.85** | 0.41 | 0.45 | 0.10 | 0.42 | 0.10 | 0.42 |
| **Node5** | **0.85** | 0.24 | 0.68 | 0.20 | 0.17 | 0.68 | 0.17 | 0.68 |
| **Node6** | **0.85** | **0.99** | 0.10 | **0.76** | 0.11 | 0.11 | 0.11 | 0.11 |
| **Node7** | **0.95** | **0.80** | **0.99** | 0.64 | **0.87** | 0.11 | 0.11 | 0.11 |
| **Node8** | **0.79** | 0.34 | **0.96** | 0.11 | 0.54 | 0.11 | 0.11 | 0.11 |
| **Node9** | **0.92** | 0.53 | **0.82** | 0.12 | **0.74** | 0.12 | 0.12 | 0.12 |
| **Node10** | 0.61 | **0.77** | **0.97** | 0.10 | 0.10 | 0.10 | 0.10 | 0.10 |
| **Node11** | **0.87** | 0.02 | **0.79** | 0.02 | 0.02 | 0.02 | 0.02 | 0.02 |
| **Node12** | **0.98** | 0.19 | 0.19 | 0.19 | 0.19 | 0.19 | 0.19 | 0.19 |
| **Node13** | **0.98** | 0.17 | 0.17 | 0.17 | 0.17 | 0.17 | 0.17 | 0.17 |
| **Node14** | 0.36 | **0.97** | 0.16 | 0.26 | **0.70** | 0.11 | 0.29 | 0.11 |
| **Node15** | 0.19 | **0.92** | 0.19 | 0.19 | 0.35 | 0.19 | 0.57 | 0.19 |
| **Node16** | 0.23 | **0.75** | 0.23 | 0.23 | 0.23 | 0.23 | **0.78** | 0.23 |
| **Node17** | 0.25 | 0.25 | 0.25 | 0.25 | 0.25 | 0.25 | **0.93** | 0.25 |
| **Node18** | 0.25 | **0.93** | 0.25 | 0.25 | 0.25 | 0.25 | 0.25 | 0.25 |
| **Node19** | 0.10 | **0.95** | 0.10 | 0.10 | 0.54 | 0.10 | 0.10 | 0.10 |
| **Node20** | 0.05 | **1.00** | 0.05 | 0.05 | 0.05 | 0.05 | 0.05 | 0.05 |
| **Node21** | 0.12 | **0.99** | 0.12 | 0.12 | 0.12 | 0.12 | 0.12 | 0.12 |
| **Node22** | 0.61 | **0.93** | 0.20 | 0.44 | **0.85** | 0.06 | 0.06 | 0.06 |
| **Node23** | 0.08 | **0.97** | 0.08 | 0.42 | 0.08 | 0.08 | 0.08 | 0.08 |
| **Node24** | 0.07 | **1.00** | 0.07 | 0.16 | 0.07 | 0.07 | 0.07 | 0.07 |
| **Node25** | 0.23 | **0.93** | 0.23 | 0.31 | 0.23 | 0.23 | 0.23 | 0.23 |
| **Node26** | 0.00 | **1.00** | 0.00 | 0.00 | 0.00 | 0.00 | 0.00 | 0.00 |
| **Node27** | 0.06 | **1.00** | 0.06 | 0.06 | 0.06 | 0.06 | 0.06 | 0.06 |
| **Node28** | 0.07 | **1.00** | 0.07 | 0.07 | 0.07 | 0.07 | 0.07 | 0.07 |
| **Node29** | 0.01 | **1.00** | 0.01 | 0.01 | 0.01 | 0.01 | 0.01 | 0.01 |
| **Node30** | 0.02 | **1.00** | 0.02 | 0.02 | 0.02 | 0.02 | 0.02 | 0.02 |
| **Node31** | 0.11 | **0.98** | 0.11 | 0.11 | 0.11 | 0.11 | 0.11 | 0.11 |
| **Node32** | 0.12 | **0.98** | 0.12 | 0.12 | 0.12 | 0.12 | 0.12 | 0.12 |
| **Node33** | 0.14 | **0.97** | 0.14 | 0.14 | 0.14 | 0.14 | 0.14 | 0.14 |
| **Node34** | **0.90** | 0.50 | 0.50 | 0.50 | **1.00** | 0.07 | 0.07 | 0.07 |
| **Node35** | **0.99** | **0.78** | **0.78** | **0.78** | **0.99** | 0.10 | 0.10 | 0.10 |
| **Node36** | 0.04 | 0.04 | 0.04 | 0.04 | **1.00** | 0.04 | 0.04 | 0.04 |
